# Considering Gender Within the Four Theoretical Domains of Subjective Age

**DOI:** 10.1093/geroni/igaa057.1826

**Published:** 2020-12-16

**Authors:** Shelbie Turner, Richard Settersten, Karen Hooker

**Affiliations:** 1 Oregon State University, Corvallis, Oregon, United States; 2 BSS, Corvallis, Oregon, United States

## Abstract

The broad construct of subjective age is informed by four theoretical domains – self-perceptions of aging, old age stereotypes, age identity, and awareness of age related change (Kotter-Gruhn, Kornadt, & Stephan, 2016). Each of the theoretical domains is distinct yet interconnected, and analyzing how gender operates within each yields a more nuanced understanding of gender’s influence on subjective age. In our presentation, we will offer a review of researchers’ consideration of gender in studies of each subjective age theoretical domain, describing (1) how gender has and has not been included, (2) key findings when gender has been included, and (3) insights into how researchers might better include – or even center – gender when studying each domain. In so doing, we highlight the contributions of past scholarship on gender and subjective age and offer insights for future studies on the topic.

